# Global stability of the endemic equilibrium of a discrete SIR epidemic model

**DOI:** 10.1186/1687-1847-2013-42

**Published:** 2013-02-25

**Authors:** Xia Ma, Yicang Zhou, Hui Cao

**Affiliations:** 1 Department of Applied Mathematics, Xi’an Jiaotong University, Xi’an, 710049 China; 2 School of Science, Shaanxi University of Science & Technology, Xi’an, 710021 China

**Keywords:** discrete SIR model, global stability, asymptotic behavior, persistence

## Abstract

The basic reproductive number $R_{0}$ of a discrete SIR epidemic model is defined and the dynamical behavior of the model is studied. It is proved that the disease free equilibrium is globally asymptotically stable if $R_{0}<1$, and the persistence of the model is obtained when $R_{0}>1$. The main attention is paid to the global stability of the endemic equilibrium. Sufficient conditions for the global stability of the endemic equilibrium are established by using the comparison principle. Numerical simulations are done to show our theoretical results and to demonstrate the complicated dynamics of the model.

**Electronic supplementary material:**

The online version of this article (doi:10.1186/1687-1847-2013-42) contains supplementary material, which is available to authorized users.

## 1 Introduction

Mathematical models have played a significant role in describing the dynamical evolution of infectious diseases. SIR model is one of the classical epidemic models with compartment structure, suitable for the transmission of infectious diseases that confer long-lasting immunity such as chicken pox and SARS. The host population is divided into three epidemiological groups: the susceptibles, the infectives, and the removed/recovered. The transmission dynamics of an infectious disease is described by modeling the population movements among those epidemiological compartments.

There is an increasing interest in the study and application of discrete epidemic models. Allen *et al.* have studied some discrete-time SI, SIR, and SIS epidemic models, compared the dynamics of deterministic and stochastic discrete-time epidemic models, and also applied the discrete epidemic model to the spread of rabies [1–3]. Allen and Driessche have given the basic reproductive number for some discrete-time epidemic models [4]. Castillo-Chavez and Yakubu have investigated a discrete SIS model with complex dynamics [5]. Zhou *et al.* have formulated and discussed the dynamical behavior of age-structured SIS models [6, 7]. A stage-structured model, a discrete epidemic model with seasonal variation in environment, a discrete tuberculosis transmission model, and many other discrete epidemic models have been constructed, studied, and applied [8–10]. One reason for the upsurge of discrete epidemic models is that discrete models have advantages in describing an infectious disease since epidemic data are usually collected in discrete time units, which would make it more convenient to use discrete-time models [11].

The studies on discrete epidemic models are relatively few compared with those on continuous epidemic models. Most research works on discrete epidemic models concern the definition of the basic reproductive number, the global stability of the disease free equilibrium, the persistence of diseases, the existence and local stability of endemic equilibria, the existence of flip bifurcation and Hopf bifurcation. The results on the global stability of the endemic equilibrium are quite few for discrete epidemic models due to the following two factors: (1) There is not enough effective theory and methods for global stability of discrete dynamical systems; (2) The discrete model exhibits much more complicated dynamical behaviors than its corresponding continuous model.

We will study the global stability of the endemic equilibrium of a discrete SIR model and get sufficient conditions. The discrete SIR model, the biological requirement of the model, the basic reproductive number, and the invariant domain of the model are given in the next section. The global stability of disease free equilibrium and the persistence are discussed in Section 3. The sufficient conditions for the global stability of endemic equilibrium are obtained in Section 4. Numerical simulations and application are presented to demonstrate our theoretical results, to show the complex dynamics and application example of the model in Section 5. Conclusions and discussions are included in the last section.

## 2 The discrete SIR model

There are different approaches to model infectious disease evolution in discrete time. The recurrent difference equations from the discretization of continuous differential equation models is one of the direct and frequently-used modeling approaches. This kind of model can be well understood in application under reasonable assumptions though there are some limitations on the range of parameters. We will adopt this approach to formulate model and assume that the infected people will obtain permanent immunity after they get recovered from infection.

Let N(t) denote the number of the host population at time *t*. According to the disease transmission mechanism, the host population is grouped into three epidemiological compartments. Let S(t), I(t), and R(t) be the number of individuals in the susceptible, infective, and removed/recovered compartments at time *t*, respectively. In addition to the death and recruitment, there are population movements among those three epidemiological compartments from time unit *t* to time t+1. We assume that the recruited individuals (by birth and immigration) are constant and enter the susceptible compartment. After one unit time, the susceptible individuals may stay in the susceptible compartment, or get infected and move to the infectious compartment, or die. The individuals in the infective compartment can keep being the infective, or get recover and transfer to the recovered compartment, or die. The individuals in the recovered compartment never leave the compartment unless they die. The discrete SIR model is

1 $$\left\{ \begin{array}{c} S(t+1)=S(t)+\Lambda -\frac{\beta S(t)I(t)}{N(t)}-\mu S(t),\\ I(t+1)=I(t)+\frac{\beta S(t)I(t)}{N(t)}-(\mu +\delta +\gamma )I(t),\\ R(t+1)=R(t)+\gamma I(t)-\mu R(t), \end{array}\right.$$

where Λ is the constant recruitment rate of the population, *β* is the transmission rate, *μ* is the natural death rate, *δ* is the death rate caused by disease, and *γ* is the recovery rate. The biological background requires that all parameters be non-negative.

There is abundant amount of research into SIR models since they capture the basic evolution mechanism of the infectious diseases when the recovered individuals will acquire life-long immunity. A lot of results on the existence and global stability of the endemic equilibrium have been obtained for continuous SIR models with various transmission rate. However, the result on the global stability of the endemic equilibrium for discrete SIR model is rare, even for the model with mass action incidence. We will try to give sufficient conditions for the global stability of the endemic equilibrium. Those results on global stability of the endemic equilibrium can promote the study on this challengeable problem.

Adding all equations in (1), we see that the number of host population, N(t), satisfies

2 N(t+1)=N(t)+Λ−μN(t)−δI(t)≤Λ+(1−μ)N(t).

The equation $\tilde{N}(t+1)=\Lambda +(1-\mu )\tilde{N}(t)$ has a unique equilibrium ${\tilde{N}}^{*}=\frac{\Lambda }{\mu }$, which is globally asymptotically stable, namely, $\lim_{t\to \infty }\tilde{N}(t)={\tilde{N}}^{*}$. The comparison principle implies that $N(t)\leq \tilde{N}(t)\leq {\tilde{N}}^{*}$ when $N(0)\leq \tilde{N}(0)\leq {\tilde{N}}^{*}$. In the analysis of model (1), we assume that

3 $$0\leq S(0),\;0\leq I(0),\;0\leq R(0)\;\text{and}\;S(0)+I(0)+R(0)\leq {\tilde{N}}^{*}.$$

The epidemiological interpretation requires the solution of model (1) with initial values satisfying (3) to be non-negative. This requirement can be met if the following two inequalities hold:

4 β+μ<1,μ+δ+γ<1.

Those two conditions in (4) are natural requirements for model (1). The first inequality, β+μ<1, says that the percentage of the susceptible individuals who die or get infected is less than one within a unit time. The second inequality, μ+δ+γ<1, states that the percentage of the infected people who die or get recovered is less than one within a unit time. It is easy to verify that those two inequalities ensure S(t)≥0, I(t)≥0, and R(t)≥0 for all t≥0 if the initial values satisfy (3).

In the rest of the paper, we assume that the initial conditions and the parameters satisfy (3) and (4), respectively. It is easy to verify that the domain

$$\Omega_{1}=\left\{(S,I,R)\in R_{+}^{3}|S+I+R\leq \frac{\Lambda }{\mu }\right\}$$

is a compact, positively invariant set for model (1). $\Omega_{1}$ is also a global compact attractor of system (1) since it attracts all positive orbits with an initial value $(S_{0},I_{0},R_{0})\in R_{+}^{3}$.

The basic reproductive number, $R_{0}$, is fundamental and widely used in the study of epidemiological models. $R_{0}$ is the average number of secondary infections produced by one infected individual during the entire course of infection in a completely susceptible population. $R_{0}$ often serves as a threshold parameter that predicts whether an infection dies out or keeps persistence in a population. Following the idea and framework given in [4, 12, 13], we obtain the formula of the basic reproduction number of model (1), $R_{0}=\frac{\beta }{\mu +\delta +\gamma }$. The magnitude of $R_{0}$ plays a crucial role in determining the dynamical behavior of model (1).

## 3 Extinction and persistence for the disease

This section focuses on the disease extinction or persistence, which is determined by the stability of the disease free equilibrium and the existence of endemic equilibrium of model (1). It is obvious that $E^{0}(\frac{\Lambda }{\mu },0,0)$ is an equilibrium of model (1). $E^{0}$ is called the disease free equilibrium since *I* and *R* components are zero. The stability of the disease free equilibrium $E^{0}$ is given in the following theorem.

**Theorem 1** *The disease free equilibrium* $E^{0}$ *of model* (1) *is globally asymptotically stable if* $R_{0}<1$, *and* $E^{0}$ *is unstable if* $R_{0}>1$.

*Proof* The linearized matrix of (1) at the disease free equilibrium $E^{0}$ is

$$A=\left(\begin{array}{ccc} 1-\mu & -\beta & 0\\ 0 & 1+\beta -\mu -\delta -\gamma & 0\\ 0 & \gamma & 1-\mu \end{array}\right).$$

Obviously, the eigenvalues of matrix *A* are $\lambda_{1}=\lambda_{2}=1-\mu$ and $\lambda_{3}=1+\beta -\mu -\delta -\gamma$, respectively. The conditions β+μ<1, μ+δ+γ<1, and $R_{0}<1$ imply that $|\lambda_{i}|<1$, i=1,2,3. Therefore, the disease free equilibrium $E^{0}$ is asymptotically stable. When $R_{0}>1$, we have $\lambda_{3}=1+\beta -\mu -\delta -\gamma >1$, then the disease free equilibrium $E^{0}$ is unstable.

From the second equation of model (1) and the fact S(t)≤N(t), we have

5 $$I(t+1)\leq I(t)+\beta I(t)-(\mu +\delta +\gamma )I(t)=\left(1-(\mu +\delta +\gamma )(1-R_{0})\right)I(t).$$

The conditions μ+δ+γ<1 and $R_{0}<1$ imply that $0<1-(\mu +\delta +\gamma )(1-R_{0})<1$. The recurrent use of inequality (5) yields

6 $$I(t)={\left(1-(\mu +\delta +\gamma )(1-R_{0})\right)}^{t}I(0).$$

The inequality (6) implies that $\lim_{t\to \infty }I(t)=0$.

From the fact $\lim_{t\to \infty }I(t)=0$, we know that for any ε>0, there exists a large positive integer $T_{1}$ such that I(t)<ε when $t\geq T_{1}$. Consequently,

7 $$R(t+1)=R(t)+\gamma I(t)-\mu R(t)\leq R(t)+\gamma \varepsilon -\mu R(t)\;\text{for }t\geq T_{1}.$$

The equation $\tilde{R}(t+1)=\tilde{R}(t)+\gamma \varepsilon -\mu \tilde{R}(t)$ has a unique equilibrium ${\tilde{R}}^{*}=\frac{\gamma \varepsilon }{\mu }$, which is globally asymptotically stable. The comparison principle implies that there exists an integer $T_{2}>T_{1}$ such that $R(t)\leq \tilde{R}(t)<\frac{2\gamma \varepsilon }{\mu }$ if $t>T_{2}$. The arbitrary *ε* implies that $\lim_{t\to \infty }R(t)=0$.

From (2) we have

8 $$N(t)+\Lambda -\delta \varepsilon -\mu N(t)\leq N(t+1)\leq \Lambda +(1-\mu )N(t)\;\text{if }t>T_{1}.$$

From the left inequality of (8) and the comparison principle, we know that for any given $\varepsilon_{1}>0$, there exists an integer $T_{3}>T_{1}$ such that $N(t)\geq \frac{\Lambda -\delta \varepsilon }{\mu }-\varepsilon_{1}$ for all $t>T_{3}$. From the right inequality of (8) and the comparison principle, we know that for any given $\varepsilon_{2}>0$, there exists an integer $T_{4}>T_{1}$ such that $N(t)\leq \frac{\Lambda }{\mu }+\varepsilon_{2}$ for all $t>T_{4}$. Let $T_{5}=T_{3}+T_{4}$, then the inequalities

$$\frac{\Lambda -\delta \varepsilon }{\mu }-\varepsilon_{1}\leq N(t)\leq \frac{\Lambda }{\mu }+\varepsilon_{2}\;\text{for all }t>T_{5},$$

and the arbitrary *ε*, $\varepsilon_{1}$, and $\varepsilon_{2}$ imply that $\lim_{t\to \infty }N(t)=\frac{\Lambda }{\mu }$, *i.e.*, $\lim_{t\to \infty }S(t)=\lim_{t\to \infty }(N(t)-I(t)-R(t))=\frac{\Lambda }{\mu }$. Those limits

$$\lim_{t\to \infty }S(t)=\frac{\Lambda }{\mu },\;\lim_{t\to \infty }I(t)=0,\;\lim_{t\to \infty }R(t)=0$$

imply that the disease free equilibrium of (1) is globally asymptotically stable when $R_{0}<1$. □

$R_{0}>1$ means that the average number of a new infection by an infected individual is more than one. The epidemiological interpretation indicates that the disease may keep persistent in the population. The following theorem confirms the persistence of the disease in the case $R_{0}>1$.

**Theorem 2** *If* $R_{0}>1$, *then the disease will keep persistence in the population*, *i*.*e*., *there exists a positive* *ε* *such that the solution of model* (1) *with the initial value* I(0)>0 *satisfies* ${lim inf}_{t\to \infty }I(t)>\varepsilon$.

*Proof* We define the sets $X=\Omega_{1}=\{(S,I,R)\in R_{+}^{3}|S+I+R\leq \frac{\Lambda }{\mu }\}$, $X_{0}=\{(S,I,R)\in X|I>0,R>0\}$, and $\partial X_{0}=X\setminus X_{0}$. Let Φ:X→X, $\Phi_{t}(x^{0})=\phi (t,x^{0})$, be the solution map of model (1) with $\phi (0,x^{0})=x^{0}$ and $x^{0}=(S(0),I(0),R(0))$. Let $M=\{\Phi_{0}\}=(\frac{\Lambda }{\mu },0,0)$ and

$$M_{\partial }=\left\{(S,I,R)\in \partial X_{0}|\Phi_{t}(S,I,R)\in \partial X_{0},\forall t\geq 0\right\}.$$

It is clear that $\{(S,0,0)\in \partial X_{0}|S\geq 0\}\subset M_{\partial }$ and $M_{\partial }=\{(S,I,R)\in \partial X_{0}|I=0\}$. Furthermore, there is exactly one fixed point $\Phi_{0}$ of Φ in $M_{\partial }$. The equation

$$S_{1}(t+1)=\Lambda +(1-\mu )S_{1}(t)$$

has a positive equilibrium $S_{*}=\frac{\Lambda }{\mu }$, which is globally attractive. By using Lemma 5.9 in [14], we know that no subset of ℳ forms a cycle in $\partial X_{0}$. The fact $\Phi_{t}((S(0),0,R(0)))=(S(t),0,R(t))$ implies that $\Phi_{t}(M_{\partial })\subset M_{\partial }$. If $x^{0}=(S(0),0,R(0))\in M_{\partial }$, then we have $\lim_{t\to \infty }S(t)=\frac{\Lambda }{\mu }$, $\lim_{t\to \infty }R(t)=\lim_{t\to \infty }{\left(1-\mu \right)}^{t}R(0)=0$, and $\Omega (M_{\partial })=\Phi_{0}$.

Since 0≤I(t)≤N(t) and N(t+1)=Λ+N(t)−μN(t)−δI(t), we have N(t+1)≥N(t)+Λ−(μ+δ)N(t) and N(t+1)≤N(t)+Λ−μN(t). The difference equation $N_{1}(t+1)=\Lambda +(1-\mu -\delta )N_{1}(t)$ has a unique equilibrium $N_{1}^{*}=\frac{\Lambda }{\mu +\delta }$, and N(t+1)=N(t)+Λ−μN(t) has a unique equilibrium $N_{2}^{*}=\frac{\Lambda }{\mu }$, which is globally asymptotically stable. Therefore, for any given ε>0, there exists a $T_{1}>0$ such that $\frac{\Lambda }{\left(\mu +\delta \right)}-\varepsilon \leq N(t)\leq \frac{\Lambda }{\mu }+\varepsilon$ for all $t\geq T_{1}$.

If $R_{0}>1$, then we can prove that there exists a small positive number *σ* such that

9 $$\underset{t\to \infty }{lim sup}d\left(\Phi_{t}\left(S^{0},I^{0},R^{0}\right),\Phi_{0}\right)\geq \sigma \;\text{for }\left(S^{0},I^{0},R^{0}\right)\in X_{0}.$$

If the conclusion in (9) does not hold, then for any positive number *σ*, there exist a point $(S_{\sigma }^{0},I_{\sigma }^{0},R_{\sigma }^{0})\in X_{0}$ and a large $T_{2}>T_{1}$ such that

10 $$d\left(\Phi_{t}\left(S_{\sigma }^{0},I_{\sigma }^{0},R_{\sigma }^{0}\right),\Phi_{0}\right)<\sigma \;\text{for }t>T_{2}.$$

The inequality in (10) implies that

11 $$I(t)\leq \sigma \;\text{and}\;S(t)>\frac{\Lambda }{\mu }-\sigma \;\text{if }t>T_{2}.$$

When $t>T_{2}$, the equations in (1) imply that

12 $$\begin{array}{r} N(t+1)\leq N(t)+\Lambda -\mu N(t),\\ I(t+1)>I(t)+\frac{\beta (\frac{\Lambda }{\mu }-\sigma )I(t)}{N(t)}-(\mu +\delta +\gamma )I(t). \end{array}$$

From the first inequality in (12), we know that there exists a number $T_{3}>T_{2}$ such that $N(t)\leq \frac{\Lambda }{\mu }$ holds for all $t>T_{3}$. When $t>T_{3}$, we substitute the inequality $N(t)\leq \frac{\Lambda }{\mu }$ into the second inequality of (12) to obtain

13 $$\begin{array}{rcl} I(t+1) & > & I(t)+\frac{\beta (\Lambda -\mu \sigma )I(t)}{\Lambda }-(\mu +\delta +\gamma )I(t)\\ = & I(t)\left(1+\left(R_{0}(1-\mu \sigma /\Lambda )-1\right)(\mu +\delta +\gamma )\right). \end{array}$$

If we choose *σ* small enough, then the condition $R_{0}>1$ implies that

14 $$R_{0}(1-\mu \sigma /\Lambda )-1>0\;\text{and}\;1+\left(R_{0}(1-\mu \sigma /\Lambda )-1\right)(\mu +\gamma )>1.$$

From the inequalities in (13) and (14), we have that $\lim_{t\to \infty }I(t)=\infty$. The limit $\lim_{t\to \infty }I(t)=\infty$ contradicts the inequality I(t)<σ in (11). The contradiction comes from the assumption given in (10). Therefore, the conclusion in (9) holds true. Subsequently, $W^{s}(\Phi_{0})\cap X_{0}=\emptyset$ and $\Phi_{0}$ is isolated in *X*. From Theorem 1.3.1 and Remark 1.3.1 in [15], it follows that Φ is uniformly persistent with respect to $\left(X_{0},\partial X_{0}\right)$. Furthermore, Theorem 1.3.4 in [15] implies that the solutions of model (1) are uniformly persistent with respect to $\left(X_{0},\partial X_{0}\right)$ when $R_{0}>1$. That is, there exists an ε>0 such that ${lim inf}_{t\to \infty }I(t)>\varepsilon >0$. □

The conditions in Theorem 1 and Theorem 2 are very simple though the proof is long and complicated. It is very easy to determine whether the disease goes to extinction or keeps persistence in the population by the magnitude of the basic reproductive number, $R_{0}$. The underlining mechanism of the nice threshold result given in Theorem 1 is that the number of the infected population will gradually become lower and lower if the new infection created by an individual during his/her infection period is less than one. The epidemiological interpretation of Theorem 2 says that there is at least a certain number of infected population if the new infection created by an individual during his/her infection period is more than one. The necessary condition is to eliminate an infectious disease, to reduce $R_{0}$, and to have $R_{0}<1$. The basis reproductive number, $R_{0}$, is a very useful quantity in mathematical analysis and epidemiological application.

## 4 The stability of the endemic equilibrium

This section deals with the existence and stability of the endemic equilibrium of model (1). Let $E^{*}(S^{*},I^{*},R^{*})$ be the endemic equilibrium of model (1). Then $S^{*}$, $I^{*}$, and $R^{*}$ satisfy the following equations:

$$\Lambda -\frac{\beta S^{*}I^{*}}{N^{*}}-\mu S^{*}=0,\;\frac{\beta S^{*}}{N^{*}}-(\mu +\delta +\gamma )=0,\;\gamma I^{*}-\mu R^{*}=0,$$

where $N^{*}=S^{*}+I^{*}+R^{*}$. By straightforward and careful calculations, we know that model (1) has a unique endemic equilibrium when $R_{0}>1$ with

$$\begin{array}{c} S^{*}=\frac{\Lambda (\mu +\gamma )}{\mu ((\mu +\delta +\gamma )(R_{0}-1)+(\mu +\gamma ))},\\ I^{*}=\frac{\Lambda (R_{0}-1)}{\left(\mu +\delta +\gamma )(R_{0}-1)+(\mu +\gamma \right)},\;R^{*}=\frac{\gamma I^{*}}{\mu }. \end{array}$$

The local stability of the endemic equilibrium $E^{*}$ is given in the following theorem.

**Theorem 3** *If* $R_{0}>1$, *then the endemic equilibrium* $E^{*}=(S^{*},I^{*},R^{*})$ *of model* (1) *is locally asymptotically stable*.

*Proof* In order to discuss the stability of the endemic equilibrium of model (1), we consider the following equivalent system:

15 $$\left\{ \begin{array}{c} N(t+1)=N(t)+\Lambda -\mu N(t)-\delta I(t),\\ I(t+1)=I(t)+(\mu +\delta +\gamma )(R_{0}-1)I(t)-\frac{\beta I{\left(t\right)}^{2}}{N(t)}-\frac{\beta I(t)R(t)}{N(t)},\\ R(t+1)=R(t)+\gamma I(t)-\mu R(t). \end{array}\right.$$

When $R_{0}>1$, the positive equilibrium of system (15) is $\left(N^{*},I^{*},R^{*}\right)$, where

$$N^{*}=\frac{\Lambda (\mu +\gamma )R_{0}}{\mu ((\mu +\delta +\gamma )(R_{0}-1)+(\mu +\gamma ))}.$$

The linearization matrix of (15) at the positive equilibrium $\left(N^{*},I^{*},R^{*}\right)$ is

$$A_{1}=\left(\begin{array}{ccc} 1-\mu & -\delta & 0\\ \frac{\beta I^{*}(I^{*}+R^{*})}{{N^{*}}^{2}} & 1-\frac{\beta I^{*}}{N^{*}} & -\frac{\beta I^{*}}{N^{*}}\\ 0 & \gamma & 1-\mu \end{array}\right).$$

The characteristic equation of matrix $A_{1}$ is

$$\phi (\lambda )=(1-\mu -\lambda )\left((1-\mu -\lambda )\left(1-\frac{\beta I^{*}}{N^{*}}-\lambda \right)+\frac{\gamma \beta I^{*}}{N^{*}}+\frac{\delta \beta I^{*}(I^{*}+R^{*})}{{N^{*}}^{2}}\right).$$

We see that the equation ϕ(λ)=0 has an eigenvalue $0<\lambda_{1}=1-\mu <1$. Therefore, in order to determine the stability of the positive equilibrium of model (15), we discuss the roots of the following equation:

$$\begin{array}{rcl} \phi_{1}(\lambda ) & = & (1-\mu -\lambda )\left(1-\frac{\beta I^{*}}{N^{*}}-\lambda \right)+\frac{\gamma \beta I^{*}}{N^{*}}+\frac{\delta \beta I^{*}(I^{*}+R^{*})}{{N^{*}}^{2}}\\ = & \lambda^{2}-\left(2-\mu -\frac{\beta I^{*}}{N^{*}}\right)\lambda +(1-\mu )\left(1-\frac{\beta I^{*}}{N^{*}}\right)+\frac{\gamma \beta I^{*}}{N^{*}}+\frac{\delta \beta I^{*}(I^{*}+R^{*})}{{N^{*}}^{2}}. \end{array}$$

When $R_{0}>1$, the calculation yields

$$\begin{array}{c} \begin{array}{rl} \phi_{1}(1) & =1-\left(2-\mu -\frac{\beta I^{*}}{N^{*}}\right)+(1-\mu )\left(1-\frac{\beta I^{*}}{N^{*}}\right)+\frac{\gamma \beta I^{*}}{N^{*}}+\frac{\delta \beta I^{*}(I^{*}+R^{*})}{{N^{*}}^{2}}\\ =\frac{\mu \beta I^{*}}{N^{*}}+\frac{\gamma \beta I^{*}}{N^{*}}+\frac{\delta \beta I^{*}(I^{*}+R^{*})}{{N^{*}}^{2}}>0, \end{array}\\ \begin{array}{rl} \phi_{1}(-1) & =1+\left(2-\mu -\frac{\beta I^{*}}{N^{*}}\right)+(1-\mu )\left(1-\frac{\beta I^{*}}{N^{*}}\right)+\frac{\gamma \beta I^{*}}{N^{*}}+\frac{\delta \beta I^{*}(I^{*}+R^{*})}{{N^{*}}^{2}}\\ =2\left(1-\mu -\frac{\beta I^{*}}{N^{*}}\right)+2+\frac{\mu \beta I^{*}}{N^{*}}+\frac{\gamma \beta I^{*}}{N^{*}}+\frac{\delta \beta I^{*}(I^{*}+R^{*})}{{N^{*}}^{2}}\\ >2(1-\mu -\beta )+2+\frac{\mu \beta I^{*}}{N^{*}}+\frac{\gamma \beta I^{*}}{N^{*}}+\frac{\delta \beta I^{*}(I^{*}+R^{*})}{{N^{*}}^{2}}>0. \end{array} \end{array}$$

Furthermore, the constant term satisfies

$$\begin{array}{rcl} c & = & (1-\mu )\left(1-\frac{\beta I^{*}}{N^{*}}\right)+\frac{\gamma \beta I^{*}}{N^{*}}+\frac{\delta \beta I^{*}(I^{*}+R^{*})}{{N^{*}}^{2}}\\ = & 1-\mu -\frac{\beta I^{*}}{N^{*}}+\frac{\gamma \beta I^{*}}{N^{*}}+\frac{\delta \beta I^{*}}{N^{*}}\frac{R_{0}-1}{R_{0}}\\ < & 1-\mu -(1-\mu -\delta -\gamma )\frac{\beta I^{*}}{N^{*}}<1. \end{array}$$

The Jury criterion [16] implies that the two roots, $\lambda_{2}$ and $\lambda_{3}$, of the equation $\phi_{1}(\lambda )=0$ satisfy $|\lambda_{2}|<1$ and $|\lambda_{3}|<1$. The linearization theory implies that the positive equilibrium $\left(N^{*},I^{*},R^{*}\right)$ of system (15) is locally asymptotically stable if $R_{0}>1$, *i.e.*, the endemic equilibrium $E^{*}$ of system (1) is locally asymptotically stable. □

The global stability of the endemic equilibrium $E^{*}$ of model (1) is quite difficult. Sufficient conditions are presented in two theorems for the special case δ=0 and for the general case.

**Theorem 4** *Assume that* δ=0. *The endemic equilibrium* $E^{*}(S^{*},I^{*},R^{*})$ *of model* (1) *is globally asymptotically stable if* $R_{0}>\frac{\mu +\gamma }{\mu }$ *and* γ<μ.

*Proof* When δ=0, the host population N(t)=S(t)+I(t)+R(t) satisfies

16 $$N(t+1)=\Lambda +(1-\mu )N(t),\;N(0)=N_{0}>0,$$

which has the solution

$$N(t)=\frac{\Lambda (1-{\left(1-\mu \right)}^{t})}{\mu }+{\left(1-\mu \right)}^{t}N_{0}.$$

From the solution of the host population, we have that $\lim_{t\to \infty }N(t)=N^{*}=\frac{\Lambda }{\mu }$, and the limit of N(t) is independent of the initial value $N_{0}$.

The global stability of the endemic equilibrium, $E^{*}$, of model (1) is equivalent to the global stability of the endemic equilibrium $E_{N}^{*}(N^{*},S^{*},I^{*})$ of the following model:

17 $$\left\{ \begin{array}{c} N(t+1)=\Lambda +(1-\mu )N(t),\\ S(t+1)=S(t)+\Lambda -\frac{\beta S(t)I(t)}{N(t)}-\mu S(t),\\ I(t+1)=I(t)+\frac{\beta S(t)I(t)}{N(t)}-(\mu +\gamma )I(t). \end{array}\right.$$

The first equation of model (17) is independent of other two state variables S(t) and I(t). The fact that $\lim_{t\to \infty }N(t)=N^{*}$ leads to the following limit model:

18 $$\left\{ \begin{array}{c} S(t+1)=S(t)+\Lambda -\frac{\beta \mu S(t)I(t)}{\Lambda }-\mu S(t),\\ I(t+1)=I(t)+\frac{\beta \mu S(t)I(t)}{\Lambda }-(\mu +\gamma )I(t). \end{array}\right.$$

The limiting model (18) possesses the same dynamical property as that of model (17). Both equations of model (18) are nonlinear, but with a similar term $\frac{\beta \mu S(t)I(t)}{\Lambda }$. We introduce the new variable L(t)=S(t)+I(t) to have one linear equation, then we have

19 $$\begin{array}{r} L(t+1)=L(t)+\Lambda -\gamma I(t)-\mu L(t),\\ I(t+1)=I(t)+\frac{\beta \mu (L(t)-I(t))I(t)}{\Lambda }-(\mu +\gamma )I(t). \end{array}$$

The global stability of the positive equilibrium of model (19) is equivalent to that of model (17). From the inequality 0≤I(t)≤L(t) and the first equation of (19), we have

20 $$\begin{array}{r} L(t+1)\leq L(t)+\Lambda -\mu L(t),\\ L(t+1)\geq L(t)+\Lambda -(\mu +\gamma )L(t). \end{array}$$

From those two inequalities in (20) and the comparison theorem [17], we know that for any small ε>0, there exists a positive integer $T_{1l}$ such that $L_{1}^{l}\leq L(t)\leq L_{1}^{m}$ for $t>T_{1l}$, where $L_{1}^{l}=\frac{\Lambda }{\mu +\gamma }-\varepsilon$ and $L_{1}^{m}=\frac{\Lambda }{\mu }+\varepsilon$.

When $t\geq T_{1l}$, we substitute $L_{1}^{l}\leq L(t)\leq L_{1}^{m}$ into the second equation of (19) to obtain

21 $$\begin{array}{r} I(t+1)\leq I(t)+\frac{\beta \mu (L_{1}^{m}-I(t))I(t)}{\Lambda }-(\mu +\gamma )I(t),\\ I(t+1)\geq I(t)+\frac{\beta \mu (L_{1}^{l}-I(t))I(t)}{\Lambda }-(\mu +\gamma )I(t). \end{array}$$

We consider the following two difference equations corresponding to the inequalities in (21):

22 $$\left\{ \begin{array}{c} I_{1}^{m}(t+1)=I_{1}^{m}(t)+\frac{\beta \mu (L_{1}^{m}-I_{1}^{m}(t))I_{1}^{m}(t)}{\Lambda }-(\mu +\gamma )I_{1}^{m}(t),\\ I_{1}^{l}(t+1)=I_{1}^{l}(t)+\frac{\beta \mu (L_{1}^{l}-I_{1}^{l}(t))I_{1}^{l}(t)}{\Lambda }-(\mu +\gamma )I_{1}^{l}(t). \end{array}\right.$$

We substitute $I_{1}^{m}(t)=\frac{\Lambda +\beta \mu L_{1}^{m}-\Lambda (\mu +\gamma )}{\beta \mu }x_{1}^{m}(t)$ and $I_{1}^{l}(t)=\frac{\Lambda +\beta \mu L_{1}^{l}-\Lambda (\mu +\gamma )}{\beta \mu }x_{1}^{l}(t)$ into the first and the second equations of (22) to have

23 $$\left\{ \begin{array}{cc} x_{1}^{m}(t+1)=r_{1}^{m}x_{1}^{m}(t)(1-x_{1}^{m}(t)), & \text{with }r_{1}^{m}=1-(\mu +\gamma )+\frac{\beta \mu L_{1}^{m}}{\Lambda },\\ x_{1}^{l}(t+1)=r_{1}^{l}x_{1}^{l}(t)(1-x_{1}^{l}(t)), & \text{with }r_{1}^{l}=1-(\mu +\gamma )+\frac{\beta \mu L_{1}^{l}}{\Lambda }. \end{array}\right.$$

The variables $x_{1}^{m}(t)$ and $x_{1}^{l}(t)$ satisfy the quadratic difference equation. The well-known result of the famous population model x(t+1)=rx(t)(1−x(t)) says that $x_{0}^{*}=0$ is the unique and globally asymptotically stable equilibrium if 0<r<1, whereas $x_{1}^{*}=1-\frac{1}{r}$ is the unique positive equilibrium if 1<r<3 and $x_{1}^{*}$ is globally asymptotically stable. The result on the quadratic population model x(t+1)=rx(t)(1−x(t)) implies that the first equation in (22) has a positive equilibrium $I_{1*}^{m}=L_{1}^{m}-\frac{\Lambda }{\mu R_{0}}$, which is globally asymptotically stable. A similar argument implies that the second equation in (22) has a positive equilibrium $I_{1*}^{l}=L_{1}^{l}-\frac{\Lambda }{\mu R_{0}}$, which is globally asymptotically stable if $R_{0}>\frac{\mu +\gamma }{\mu }$.

The asymptotical stability and the comparison theory imply that there exists a $T_{1i}\geq T_{1l}$ such that $I_{1}^{l}\leq I(t)\leq I_{1}^{m}$ for all $t>T_{1i}$, where $I_{1}^{l}=I_{1*}^{l}-\varepsilon$ and $I_{1}^{m}=I_{1*}^{m}+\varepsilon$. When $t\geq T_{1i}$, we substitute the inequality $I_{1}^{l}\leq I(t)\leq I_{1}^{m}$ into the first equation of (19) and have

24 $$\begin{array}{r} L(t+1)\leq L(t)+\Lambda -\mu L(t)-\gamma I_{1}^{l},\\ L(t+1)\geq L(t)+\Lambda -\mu L(t)-\gamma I_{1}^{m}. \end{array}$$

From (24) and a similar argument, we can obtain that there exists a positive integer $T_{2l}$ such that $L_{2}^{l}\leq L(t)\leq L_{2}^{m}$ for all $t\geq T_{2l}$, where $L_{2}^{l}=\frac{\Lambda -\gamma I_{1}^{m}}{\mu }-\varepsilon$, $L_{2}^{m}=\frac{\Lambda -\gamma I_{1}^{l}}{\mu }+\varepsilon$, and $L_{1}^{l}<L_{2}^{l}<L_{2}^{m}<L_{1}^{m}$.

When $t\geq T_{2l}$, the inequality $L_{2}^{l}\leq L(t)\leq L_{2}^{m}$ and the second equation of (19) imply that

25 $$\begin{array}{r} I(t+1)\leq I(t)+\frac{\beta \mu (L_{2}^{m}-I(t))I(t)}{\Lambda }-(\mu +\gamma )I(t),\\ I(t+1)\geq I(t)+\frac{\beta \mu (L_{2}^{l}-I(t))I(t)}{\Lambda }-(\mu +\gamma )I(t). \end{array}$$

A similar argument implies that there exists a $T_{2i}\geq T_{2l}$ such that $I_{2}^{l}\leq I(t)\leq I_{2}^{m}$ for all $t>T_{2i}$, where $I_{2}^{l}=L_{2}^{l}-\frac{\Lambda }{\mu R_{0}}-\varepsilon$ and $I_{2}^{m}=L_{2}^{m}-\frac{\Lambda }{\mu R_{0}}+\varepsilon$. After substituting $L_{2}^{m}=\frac{\Lambda -\gamma I_{1}^{l}}{\mu }+\varepsilon$ and $L_{2}^{l}=\frac{\Lambda -\gamma I_{1}^{m}}{\mu }-\varepsilon$ into the equations $I_{2}^{m}=L_{2}^{m}-\frac{\Lambda }{\mu R_{0}}+\varepsilon$ and $I_{2}^{l}=L_{2}^{l}-\frac{\Lambda }{\mu R_{0}}-\varepsilon$, we have

26 $$\left\{ \begin{array}{c} I_{2}^{m}=-\frac{\gamma I_{1}^{l}}{\mu }+\frac{\Lambda (R_{0}-1)}{R_{0}\mu }+2\varepsilon ,\\ I_{2}^{l}=-\frac{\gamma I_{1}^{m}}{\mu }+\frac{\Lambda (R_{0}-1)}{R_{0}\mu }-2\varepsilon . \end{array}\right.$$

Equations in (26) hold when $t>T_{2i}$ and $I_{2}^{l}\leq I(t)\leq I_{2}^{m}$. From the mathematical induction, we know that there exist sequences $T_{kl}$, $T_{ki}$, $L_{k}^{l}$, $L_{k}^{m}$, $I_{k}^{l}$, and $I_{k}^{m}$ such that $I_{k}^{l}<I(t)<I_{k}^{m}$ for all $t>T_{ki}$. Furthermore, $I_{k}^{l}$ and $I_{k}^{m}$ satisfy the following recurrence equations:

27 $$\left\{ \begin{array}{c} I_{k+1}^{m}=-\frac{\gamma I_{k}^{l}}{\mu }+\frac{\Lambda (R_{0}-1)}{R_{0}\mu }+2\varepsilon ,\\ I_{k+1}^{l}=-\frac{\gamma I_{k}^{m}}{\mu }+\frac{\Lambda (R_{0}-1)}{R_{0}\mu }-2\varepsilon . \end{array}\right.$$

(27) is a linear system of difference equations. System (27) has a positive equilibrium $P_{*}^{i}(I_{*}^{l}(\varepsilon ),I_{*}^{m}(\varepsilon ))$, where

28 $$\begin{array}{r} I_{*}^{l}(\varepsilon )=\frac{\mu \Lambda -\Lambda \gamma +\frac{\Lambda (\gamma^{2}-\mu^{2})}{\beta }-2\varepsilon \mu^{2}-2\varepsilon \mu \gamma }{\mu^{2}-\gamma^{2}},\\ I_{*}^{m}(\varepsilon )=\frac{\Lambda -\gamma I_{*}^{l}(\varepsilon )}{\mu }-\frac{\Lambda (\mu +\gamma )}{\beta \mu }+2\varepsilon . \end{array}$$

Let $\lambda_{1}$ and $\lambda_{2}$ be two roots of the linearized matrix of system (27) at the equilibrium $P_{*}^{i}$. It is easy to see that $|\lambda_{1}|=|\lambda_{2}|=\frac{\gamma }{\mu }<1$ if γ<μ. The stability theory of difference equations implies that the equilibrium $P_{*}^{i}$ of (27) is globally asymptotically stable, *i.e.*, $\lim_{k\to \infty }I_{k}^{l}=I_{*}^{l}(\varepsilon )$ and $\lim_{k\to \infty }I_{k}^{m}=I_{*}^{m}(\varepsilon )$. From the expressions of $I_{*}^{l}(\varepsilon )$ and $I_{*}^{m}(\varepsilon )$, we have that $\lim_{\varepsilon \to 0}I_{*}^{l}(\varepsilon )=\lim_{\varepsilon \to 0}I_{*}^{m}(\varepsilon )=\frac{\Lambda (R_{0}-1)}{\beta }$. From the inequality $I_{k}^{l}<I(t)<I_{k}^{m}$ and those limits, we obtain that $\lim_{t\to \infty }I(t)=\frac{\Lambda (R_{0}-1)}{\beta }$.

Similarly, we can prove that the sequences $\left\{L_{k}^{l}\right\}$ and $\left\{L_{k}^{m}\right\}$ satisfy a linear system of difference equations. The sequences $\left\{L_{k}^{l}\right\}$ and $\left\{L_{k}^{m}\right\}$ tend to the same limit when *k* tends to infinity and *ε* tends to zero, *i.e.*,

$$\lim_{k\to \infty }L_{k}^{l}=\lim_{k\to \infty }L_{k}^{m}=\frac{\Lambda (\beta \mu +\gamma (\mu +\gamma ))}{\mu (\mu +\gamma )\beta }\;\text{as }\varepsilon \to 0.$$

The inequality $L_{k}^{l}<L(t)<L_{k}^{m}$ and the limit imply $\lim_{t\to \infty }L(t)=\frac{\Lambda (\beta \mu +\gamma (\mu +\gamma ))}{\mu (\mu +\gamma )\beta }$. Finally, we have

$$\begin{array}{c} \lim_{t\to \infty }S(t)=\lim_{t\to \infty }\left(L(t)-I(t)\right)=\frac{\Lambda (\mu +\gamma )}{\mu \beta }\;\text{and}\\ \lim_{t\to \infty }R(t)=\lim_{t\to \infty }\left(N(t)-L(t)\right)=\frac{\Lambda \gamma (R_{0}-1)}{\mu \beta }. \end{array}$$

Therefore, the endemic equilibrium of system (18) is globally asymptotic stable when $R_{0}>\frac{\mu +\gamma }{\mu }$ and γ<μ. Equivalently, the endemic equilibrium of system (1) is globally asymptotic stable. □

The comparison principle is the main idea to prove Theorem 4. The limitation of the method and the construction of the comparison equations may lead to the imposed conditions $R_{0}>\frac{\mu +\gamma }{\mu }$ and γ≤μ. We do hope that the global stability of the endemic equilibrium can be proved under the condition $R_{0}>1$. When δ>0, the global stability condition of endemic equilibrium is more complicated. We use the same idea to get the sufficient stability conditions.

**Theorem 5** *If* μ>δ+γ, $\mu^{2}>\frac{(\mu +\delta ){\left(\mu +\delta +\gamma \right)}^{2}}{2+\mu +\delta +\gamma }$ *and*

$$\max\left\{\frac{\mu +\delta +\gamma }{\mu },\frac{\delta }{\mu -\delta -\gamma }\right\}<R_{0}<\left(1+\frac{2}{\mu +\delta +\gamma }\right)\frac{\mu }{\mu +\delta },$$

*then the endemic equilibrium* $E^{*}(S^{*},I^{*},R^{*})$ *of model* (1) *is globally asymptotically stable*.

*Proof* Let L(t)=S(t)+I(t) and N(t)=L(t)+R(t). We consider the equivalent model 29

The definitions of L(t) and N(t) show that 0≤I(t)≤L(t)≤N(t). The global stability of endemic equilibrium (29) is equivalent to that of model (1). From the first equation of system (29), we have

30 $$\begin{array}{r} N(t+1)\leq N(t)+\Lambda -\mu N(t),\\ N(t+1)\geq N(t)+\Lambda -(\mu +\delta )N(t). \end{array}$$

From (30) and the comparison theorem, we know that for any small ε>0, there exists a positive integer $T_{1n}$ such that $N_{1}^{l}\leq N(t)\leq N_{1}^{m}$ for all $t>T_{1n}$, where $N_{1}^{l}=\frac{\Lambda }{\mu +\delta }-\varepsilon$ and $N_{1}^{m}=\frac{\Lambda }{\mu }+\varepsilon$. From the second equation of system (29), we have

31 $$\begin{array}{r} L(t+1)\leq L(t)+\Lambda -\mu L(t),\\ L(t+1)\geq L(t)+\Lambda -(\mu +\delta +\gamma )L(t). \end{array}$$

From (31) and the comparison theorem [17], we know that there exists a positive integer $T_{1l}$ such that $L_{1}^{l}\leq L(t)\leq L_{1}^{m}$ for all $t>T_{1l}$, where $L_{1}^{l}=\frac{\Lambda }{\mu +\gamma +\delta }-\varepsilon$ and $L_{1}^{m}=\frac{\Lambda }{\mu }+\varepsilon$.

When $t>T_{1c}=\max\{T_{1n},T_{1l}\}$, the inequalities $N_{1}^{l}\leq N(t)\leq N_{1}^{m}$, $L_{1}^{l}\leq L(t)\leq L_{1}^{m}$, and the third equation of (29) can yield

32 $$\begin{array}{r} I(t+1)\leq I(t)+\beta \frac{L_{1}^{m}-I(t)}{N_{1}^{l}}I(t)-(\mu +\delta +\gamma )I(t),\\ I(t+1)\geq I(t)+\beta \frac{L_{1}^{l}-I(t)}{N_{1}^{m}}I(t)-(\mu +\delta +\gamma )I(t). \end{array}$$

The comparison equations corresponding to those inequalities in (32),

33 $$\begin{array}{r} I_{1}^{m}(t+1)=I_{1}^{m}(t)+\beta \frac{L_{1}^{m}-I_{1}^{m}(t)}{N_{1}^{l}}I_{1}^{m}(t)-(\mu +\delta +\gamma )I_{1}^{m}(t),\\ I_{1}^{l}(t+1)=I_{1}^{l}(t)+\beta \frac{L_{1}^{l}-I_{1}^{l}(t)}{N_{1}^{m}}I_{1}^{l}(t)-(\mu +\delta +\gamma )I_{1}^{l}(t) \end{array}$$

are the quadratic difference equations of the form x(t+1)=rx(t)(1−x(t)). When $\mu^{2}>\frac{(\mu +\delta ){\left(\mu +\delta +\gamma \right)}^{2}}{2+\mu +\delta +\gamma }$ and $\frac{\mu +\delta +\gamma }{\mu }<R_{0}<(1+\frac{2}{\mu +\delta +\gamma })\frac{\mu }{\mu +\delta }$, the well-know result on the population model x(t+1)=rx(t)(1−x(t)) and the comparison theorem imply that there exists an integer $T_{1i}>T_{1c}$ such that $I_{1}^{l}\leq I(t)\leq I_{1}^{m}$ for all $t>T_{1i}$, where $I_{1}^{l}=L_{1}^{l}-\frac{N_{1}^{m}}{R_{0}}-\varepsilon$ and $I_{1}^{m}=L_{1}^{m}-\frac{N_{1}^{l}}{R_{0}}+\varepsilon$.

When $t\geq T_{1i}$, we use the inequality $I_{1}^{l}\leq I(t)\leq I_{1}^{m}$ in the first two equations of system (29) to get

34 $$\begin{array}{r} N(t+1)\leq N(t)+\Lambda -\mu N(t)-\delta I_{1}^{l},\\ N(t+1)\geq N(t)+\Lambda -\mu N(t)-\delta I_{1}^{m},\\ L(t+1)\leq L(t)+\Lambda -\mu L(t)-(\delta +\gamma )I_{1}^{l},\\ L(t+1)\geq L(t)+\Lambda -\mu L(t)-(\delta +\gamma )I_{1}^{m}. \end{array}$$

A similar argument implies that there exists a positive integer $T_{2c}$ such that $N_{2}^{l}\leq N(t)\leq N_{2}^{m}$ and $L_{2}^{l}\leq L(t)\leq L_{2}^{m}$ hold for $t>T_{2c}$, where $N_{2}^{l}=\frac{\Lambda -\delta I_{1}^{m}}{\mu }-\varepsilon$, $N_{2}^{m}=\frac{\Lambda -\delta I_{1}^{l}}{\mu }+\varepsilon$, $L_{2}^{l}=\frac{\Lambda -(\gamma +\delta )I_{1}^{m}}{\mu }-\varepsilon$, $L_{2}^{m}=\frac{\Lambda -(\gamma +\delta )I_{1}^{l}}{\mu }+\varepsilon$, $N_{1}^{l}<N_{2}^{l}<N_{2}^{m}<N_{1}^{m}$, and $L_{1}^{l}<L_{2}^{l}<L_{2}^{m}<L_{1}^{m}$. When $t>T_{2c}$, we use those estimates of N(t) and L(t) in the third equation of (29) and obtain the following inequalities:

35 $$\begin{array}{r} I(t+1)\leq I(t)+\beta \frac{L_{2}^{m}-I(t)}{N_{2}^{l}}I(t)-(\mu +\delta +\gamma )I(t),\\ I(t+1)\geq I(t)+\beta \frac{L_{2}^{l}-I(t)}{N_{2}^{m}}I(t)-(\mu +\delta +\gamma )I(t). \end{array}$$

When $\mu^{2}>\frac{(\mu +\delta ){\left(\mu +\delta +\gamma \right)}^{2}}{2+\mu +\delta +\gamma }$ and $\frac{\mu +\delta +\gamma }{\mu }<R_{0}<(1+\frac{2}{\mu +\delta +\gamma })\frac{\mu }{\mu +\delta }$, a similar procedure as aforementioned can imply that there exists an integer $T_{2i}>T_{2c}$ such that $I_{2}^{l}\leq I(t)\leq I_{2}^{m}$ for all $t>T_{2i}$, where $I_{2}^{l}=L_{2}^{l}-\frac{N_{2}^{m}}{R_{0}}-\varepsilon$ and $I_{2}^{m}=L_{2}^{m}-\frac{N_{2}^{l}}{R_{0}}+\varepsilon$.

By using the mathematical induction, we obtain the sequences $T_{ki}$, $N_{k}^{l}$, $N_{k}^{m}$, $L_{k}^{l}$, $L_{k}^{m}$, $I_{k}^{l}$, and $I_{k}^{m}$ such that

$$N_{k}^{l}\leq N(t)\leq N_{k}^{m},\;L_{k}^{l}\leq L(t)\leq L_{k}^{m},\;I_{k}^{l}\leq I(t)\leq I_{k}^{m},\;t>T_{ik}.$$

Furthermore, $I_{k}^{l}$ and $I_{k}^{m}$ satisfy the following recurrence equations:

36 $$\begin{array}{r} I_{k+1}^{m}=-\frac{\delta +\gamma }{\mu }I_{k}^{l}+\frac{\delta }{R_{0}\mu }I_{k}^{m}+\frac{\Lambda (R_{0}-1)}{R_{0}\mu }+\frac{\varepsilon }{R_{0}}+2\varepsilon ,\\ I_{k+1}^{l}=-\frac{\delta +\gamma }{\mu }I_{k}^{m}+\frac{\delta }{R_{0}\mu }I_{k}^{l}+\frac{\Lambda (R_{0}-1)}{R_{0}\mu }-\frac{\varepsilon }{R_{0}}-2\varepsilon . \end{array}$$

(36) is a linear system of difference equations. Let $z(k)={\left(I_{k}^{m},I_{k}^{l}\right)}^{\tau }$, $b={\left(\frac{\Lambda (R_{0}-1)}{R_{0}\mu }+\frac{\varepsilon }{R_{0}}+2\varepsilon ,\frac{\Lambda (R_{0}-1)}{R_{0}\mu }+\frac{\varepsilon }{R_{0}}+2\varepsilon \right)}^{\tau }$, and

$$B=\left(\begin{array}{cc} \frac{\delta }{R_{0}\mu } & -\frac{\delta +\gamma }{\mu }\\ -\frac{\delta +\gamma }{\mu } & \frac{\delta }{R_{0}\mu } \end{array}\right),$$

then system (36) becomes

37 $$z(k+1)=Bz(k)+b=B^{k-1}b+\cdots +Bb+b+B^{k}z(1).$$

If the conditions of Theorem 5 hold, then we know that the two eigenvalues of matrix *B* satisfy $|\lambda_{j}|<1$ (j=1,2), and the matrix series $\sum_{k=0}^{\infty }B^{k}$ converges to ${\left(I-B\right)}^{-1}$. Under the conditions of Theorem 5, we can have $\lim_{k\to \infty }z(k)={\left(I-B\right)}^{-1}b$, *i.e.*, $z^{*}=(I_{*}^{m},I_{*}^{l})={\left({\left(I-B\right)}^{-1}b\right)}^{\tau }$ is the globally stable equilibrium of (37). Further calculation shows that

$$\lim_{\varepsilon \to 0}\left(I_{*}^{m},I_{*}^{l}\right)=\left(\frac{\Lambda (R_{0}-1)}{\left(\mu +\delta +\gamma )(R_{0}-1)+(\mu +\gamma \right)},\frac{\Lambda (R_{0}-1)}{\left(\mu +\delta +\gamma )(R_{0}-1)+(\mu +\gamma \right)}\right).$$

After taking ε→0, we have

$$\begin{array}{c} \lim_{k\to \infty }I_{k}^{l}=\lim_{k\to \infty }I_{k}^{m}=\frac{\Lambda (R_{0}-1)}{\left(\mu +\delta +\gamma )(R_{0}-1)+(\mu +\gamma \right)}\;\text{and}\\ \lim_{t\to \infty }I(t)=\frac{\Lambda (R_{0}-1)}{\left(\mu +\delta +\gamma )(R_{0}-1)+(\mu +\gamma \right)}=\frac{\Lambda (R_{0}-1)}{\beta -\delta }. \end{array}$$

A similar argument implies that

$$\begin{array}{c} \lim_{k\to \infty }N_{k}^{l}=\lim_{k\to \infty }N_{k}^{m}=\frac{\Lambda (\mu +\gamma )R_{0}}{\mu ((\mu +\delta +\gamma )(R_{0}-1)+(\mu +\gamma ))},\\ \lim_{t\to \infty }N(t)=\frac{\Lambda (\mu +\gamma )R_{0}}{\mu ((\mu +\delta +\gamma )(R_{0}-1)+(\mu +\gamma ))},\\ \lim_{k\to \infty }L_{k}^{l}=\lim_{k\to \infty }L_{k}^{m}=\frac{\Lambda (\mu +\gamma )+\Lambda (R_{0}-1)\mu }{\mu ((\mu +\delta +\gamma )(R_{0}-1)+(\mu +\gamma ))},\\ \lim_{t\to \infty }L(t)=\frac{\Lambda (\mu +\gamma )+\Lambda (R_{0}-1)\mu }{\mu ((\mu +\delta +\gamma )(R_{0}-1)+(\mu +\gamma ))}. \end{array}$$

Those limits lead to

$$\begin{array}{c} \lim_{t\to \infty }S(t)=\lim_{t\to \infty }\left(L(t)-I(t)\right)=\frac{\Lambda (\mu +\gamma )}{\mu ((\mu +\delta +\gamma )(R_{0}-1)+(\mu +\gamma ))}=\frac{\Lambda (\gamma +\mu )(R_{0}-1)}{\beta -\delta },\\ \lim_{t\to \infty }R(t)=\lim_{t\to \infty }\left(N(t)-L(t)\right)=\frac{\Lambda \gamma (R_{0}-1)}{\mu ((\mu +\delta +\gamma )(R_{0}-1)+(\mu +\gamma ))}=\frac{\Lambda \gamma (R_{0}-1)}{\mu (\beta -\delta )}. \end{array}$$

Therefore, the endemic equilibrium of system (1) is globally asymptotic stable when the conditions of Theorem 5 hold. □

There are some parameter values which can satisfy the conditions of Theorem 5. For example, if δ=γ=0, those conditions become μ>0, 1+μ>0, $1<R_{0}<1+\frac{2}{\mu }$. Then, for μ>0, we know that the conditions of Theorem 5 will hold if *δ* and *γ* are small enough.

## 5 Numerical simulations

In this section, we carry out numerical simulations to demonstrate our theoretical results and the complex dynamics of model (1) for several sets of parameters and initial values. We use the SIR model to simulate the mumps infection in China, too.

### 5.1 Simulations of model (1)

The simulations in this subsection demonstrate our theoretical results or the complex dynamics of the model. The parameter values for those simulations are selected mathematically to let the model exhibit required dynamics.

**Figure 1:**
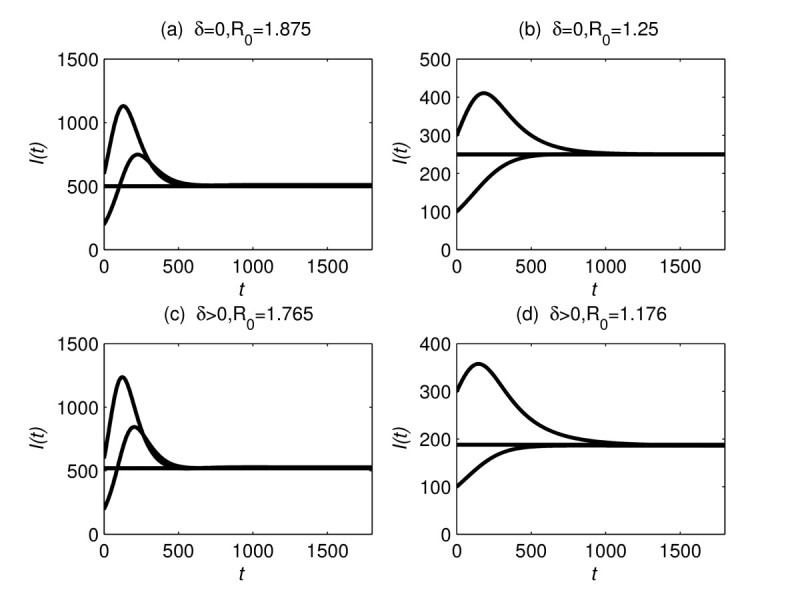
**The global stability of the endemic equilibrium of model** (**1**)**.**

When δ=0, we choose μ=0.01, γ=0.006, Λ=20, and β=0.03, then γ<μ, $\frac{\mu +\gamma }{\mu }=1.6$, and $R_{0}=1.875>1.6$. Theorem 4 implies that the endemic equilibrium $E^{*}(S^{*},I^{*},R^{*})$ of (1) is globally asymptotically stable. The number of the infectious individuals for two solutions with different initial values are shown in Figure 1(a). From Figure 1(a) we see that the number of infected people approaches its equilibrium value as *t* tends to infinity. For the same values of *δ*, *μ*, *γ*, and Λ, the straightforward calculation yields $R_{0}\in (1,1.6)$ if β∈(0.016,0.0256), which does not satisfy the condition $R_{0}>\frac{\mu +\gamma }{\mu }$ of Theorem 4. On the other hand, the simulation shows that the endemic equilibrium of model (1) may be globally asymptotically stable even if the condition $R_{0}>\frac{\mu +\gamma }{\mu }$ of Theorem 4 does not hold. The solution curves of the infected people for β=0.02 and two different initial values are given in Figure 1(b). Those simulations remind us that the condition $R_{0}>\frac{\mu +\gamma }{\mu }$ of Theorem 4 may not be necessary for the global stability. It is a pity that we cannot prove the global stability without the condition $R_{0}>\frac{\mu +\gamma }{\mu }$.

For the case δ>0, we choose δ=0.001, μ=0.01, γ=0.006, and Λ=20. Then we have $\max\{\frac{\delta }{\mu -\delta -\gamma },\frac{\mu +\delta +\gamma }{\mu }\}=1.7$, $(1+\frac{2}{\mu +\delta +\gamma })\frac{\mu }{\mu +\delta }=107.86$, $\mu^{2}>\frac{(\mu +\delta ){\left(\mu +\delta +\gamma \right)}^{2}}{2+\mu +\delta +\gamma }$, and μ>δ+γ. Theorem 5 implies that the endemic equilibrium $E^{*}$ is globally asymptotically stable when $1.7<R_{0}<107.86$. Figure 1(c) shows the global stability of the endemic equilibrium of model (1) for β=0.03 and $R_{0}=1.765>1$. The straightforward calculation shows that $R_{0}\in (1,1.7)$ for those parameter values and β∈(0.017,0.0289). Although $R_{0}\in (1,1.7)$ does not satisfy the conditions of Theorem 5, the numerical simulation shows that the endemic equilibrium of model (1) may still be globally asymptotically stable for $R_{0}\in (1,1.7)$. Figure 1(d) gives the number of the infected people for β=0.02 and $R_{0}=1.176$, where the condition of Theorem 5 does not hold, but the number of infected people approaches its equilibrium.

**Figure 2:**
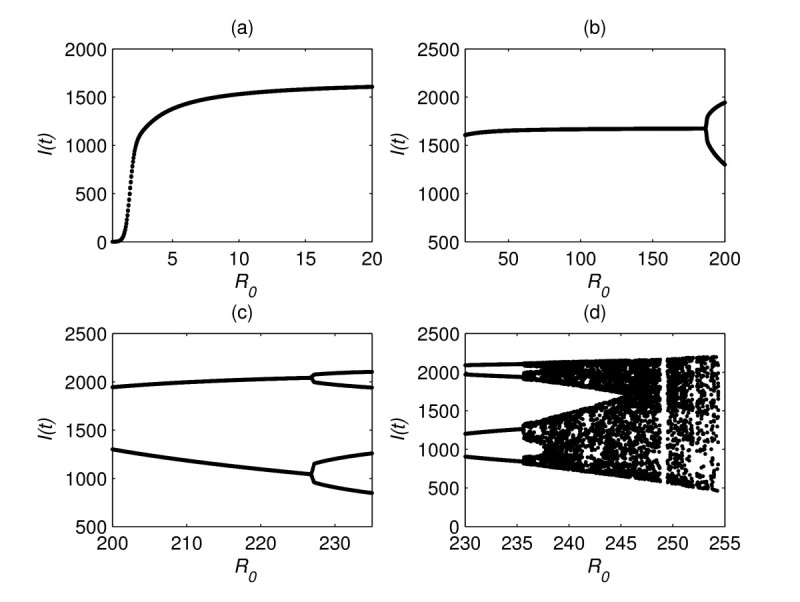
**The bifurcation diagram of the endemic equilibria and periodic solutions of model** (**1**)**.**

From the epidemiological interpretation, we assume that the inequalities 0<μ+δ+γ<1 and β+μ<1 hold. If those two inequalities hold, then all solutions of model (1) with positive initial values are non-negative, and the numerical simulations show that model (1) does not exhibit complex dynamics. If those two inequalities do not hold, then model (1) may exhibit much more complicated dynamical behaviors. The numerical simulation demonstrates that there exists a sequence of period-doubling bifurcation to chaos. Let us take μ=0.01, γ=0.001, δ=0.001, and Λ=20 to investigate the period doubling process of model (1). For small $R_{0}$ (a linear function of *β*), the endemic equilibrium is unique and globally asymptotically stable (see Figure 2(a)). When $R_{0}$ passes through 186.8, the endemic equilibrium losses its stability, and a stable periodic solution of period 2 appears (see Figure 2(b)). When $R_{0}$ passes through 226.8, the periodic solution of period 2 losses its stability, and a stable periodic solution of period four appears (see Figure 2(c)). As $R_{0}$ increases further, the periodic solution of period 4 becomes unstable, and a periodic solution of period 8 appears. The numerical simulation shows that the period-doubling bifurcation may continue and go to chaos (see Figure 2(d)).

### 5.2 Application of model (1)

The simulation in this subsection is an application of the SIR model to the mumps infection in China. The model parameters are estimated according to the demographic and epidemiological data in China from 2005 to 2010. We have compared the model simulation result of the number of annual mumps cases to the reported number of notifiable diseases.

Mumps, caused by the mumps virus, is an acute respirator infectious disease and it is spread from person to person by coughing or sneezing. Mumps usually starts with a fever and headache for a day or two. It then presents with swelling and soreness of the parotid salivary gland. The main symptom of mumps is the swollen, painful salivary glands on one or both sides of the face. The entire course of mumps infection is 10 to 14 days. The infected people will obtain permanent immunity after they get recovered from infection. The SIR model is suitable to describe the infection of mumps due to its permanent immunity. The host population is chosen to be individuals who are younger than 20 since mumps is common in children and adolescents, but rare among adults. The values of model parameters are estimated from the demographic and epidemiological data in China from 2005 to 2010.

In the application of the SIR model, the simulation time unit is taken as one day. All the parameter values will be estimated on the basis of that time unit. From the statistical yearbook of China, we know that the number of the host population (less than twenty years old) in 2005 is 359 million [18]. The annual birth rate in China from 2005 to 2010 is approximately 12/1,000, and the recruitment rate is taken as Λ=44,054 [18–21]. The annual death rate of the population is 7.06/1,000 [22, 23], hence, the daily death rate is 19.34/1,000,000. Since the age of the host population is less than twenty, an individual should be removed when his/her age is over twenty. The daily removed rate is taken to be 1/7,300, and the value of parameter *μ* is estimated to be μ=0.00001934+0.000137=0.00015634. From the fact that the entire course of mumps infection is 10 to 14 days, we take γ=1/12=0.0833 [24]. From the report on notifiable infectious diseases in China, we take δ=0.0000003.

**Figure 3:**
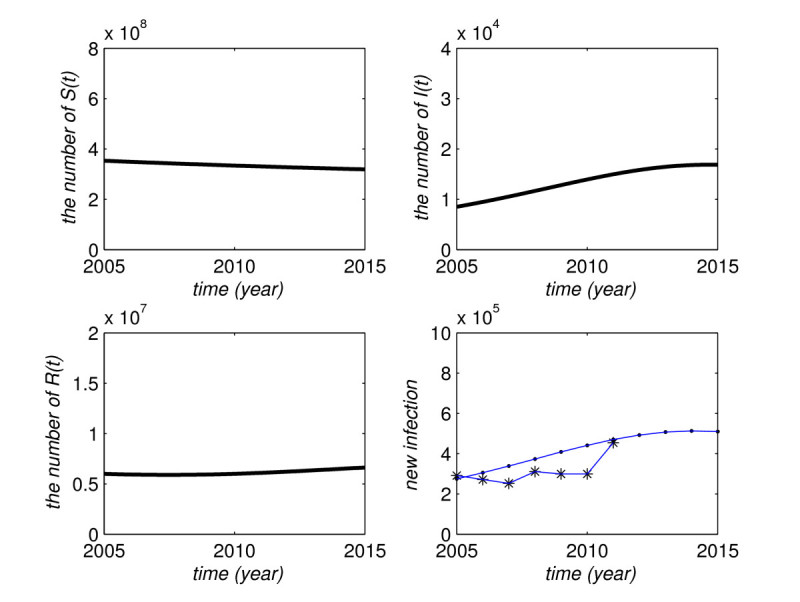
**The simulation of mumps infection in China from 2005 to 2015.**

The initial time is taken to be January first of 2005. From the data of China population and notifiable diseases in 2005 [25], the initial values are taken to be S(1)=353,117,392, I(1)=8,508, and R(1)=6,000,000, respectively. By using the method of parameter estimation in [26], we have β=0.08518. After the parameters and initial values are set, we apply the SIS model to simulate the mumps infection in China from 2005 to 2015. The simulation results are shown in Figure 3. The top left plot is the number of the susceptibles, S(t), which shows a slow downward tendency. From the demographic data in China, we know that the number of the population younger than twenty is 359 million in 2005, 320 million in 2009, and 302 million in 2010 [18, 20, 23]. The change of the host population in our simulation is consistent with the observed data. The top right is the number of the infective, I(t), which shows the slow upward tendency. The bottom left is the number of the recovered R(t). The curve in the bottom right plot is the number of new mumps infection cases from 2005 to 2015 by a model simulation. The piecewise curve with stars is the number of annually reported mumps cases out of notifiable infectious diseases from 2005 to 2011 [25]. The comparison between the model simulation and the notifiable disease report demonstrates that our model can give a good prediction for infection diseases.

## 6 Conclusion

SIR models are suitable to describe the transmission of infectious diseases with life long immunity. A lot of continuous SIR models with various transmission rates have been formulated and studied. The global stability of the endemic equilibrium of the continuous SIR model has been investigated extensively and many sufficient conditions have been obtained. Nevertheless, the discrete SIR model and its dynamics are quite few. In this paper, we have formulated a discrete SIR epidemic model and studied its asymptotical behaviors. Especially, we have got some sufficient conditions for the global stability of the endemic equilibrium. We have also applied the SIR model to the mumps infection in China. The model simulation results match the reported data of the notifiable diseases well.

Although the SIR model considered in this paper is quite simple, it exhibits very complicated dynamical behavior. The complicated dynamics of the simple SIR model reminds us that we cannot expect to have the global stability of the endemic equilibrium when $R_{0}>1$. Other conditions should be given for the global stability. In this paper we have obtained sufficient conditions for the global stability of the endemic equilibrium. Although those conditions given in our paper are not satisfactory, our result is a good exploration of the challengeable problem. We expect to prove the global stability of the endemic equilibrium when $R_{0}$ is not large. The comparison principle used in this paper and the Lyapunov function may help us to have better results.

## Authors’ original submitted files for images

Below are the links to the authors’ original submitted files for images. Authors’ original file for figure 1 Authors’ original file for figure 2 Authors’ original file for figure 3
